# A Preliminary Study on Clinical Characteristics of Patients With Autoimmune Liver Following Coronavirus Disease 2019

**DOI:** 10.1155/jotm/6675295

**Published:** 2025-10-15

**Authors:** Chenchen Yang, Yu Hu, Juanjuan Fu

**Affiliations:** ^1^School of Health and Nursing, Wuxi Taihu University, Wuxi 214064, China; ^2^Department of Pathology, The Fifth People's Hospital of Wuxi, Affiliated Wuxi Fifth Hospital of Jiangnan University, Wuxi 214000, China

**Keywords:** ANA antibody, autoimmune liver disease (AILD), COVID-19

## Abstract

**Objective:**

This study aimed to investigate the clinical features of patients with autoimmune liver disease (AILD) following coronavirus disease 2019 (COVID-19).

**Methods:**

For the AILD group, 19 inpatients who were first diagnosed with AILD were enrolled from Wuxi Fifth People's Hospital between January 2021 and December 2021. The post-COVID-19 AILD group comprised seven patients recruited after December 2022 who were initially diagnosed with COVID-19 and later diagnosed with AILD. Routine blood indices, biochemical parameters, serum-related antibodies, and serum immunoglobulin levels were assessed in the two groups. Ultrasound-guided liver biopsy was performed to observe the pathological characteristics of the liver in the two groups. The expression of immune indices in the two groups was observed by immunohistochemistry.

**Results:**

The age and calcium levels of the post-COVID-19 AILD patients were significantly lower than those of the AILD patients (*p* < 0.05). The IgM levels were significantly higher in the post-COVID-19 AILD group than in the AILD group (*p* < 0.05). No significant differences in the other routine blood and blood biochemical indices were present between the two groups. The antinuclear antibody (ANA) and SM antibody levels were compared, revealing a significantly higher percentage of ANA positivity among post-COVID-19 AILD patients than AILD patients (*p* < 0.05).

**Conclusion:**

AILD following COVID-19 has typical AILD characteristics, including acute onset, but has other characteristics that allow it to be distinguished from other AILDs.

## 1. Introduction

On May 5, 2023, the World Health Organization announced that the new coronavirus epidemic no longer constitutes a public health emergency of international concern. However, new coronavirus pneumonia cannot be ignored. Coronavirus disease 2019 (COVID-19) is caused by the pathogen new coronavirus (SARS-CoV-2). The receptor for SARS-CoV-2, angiotensin-converting enzyme 2 (ACE2) [1, 2], is expressed throughout the gastrointestinal tract and liver and may be responsible for gastrointestinal and liver involvement. ACE2 is also expressed in liver endothelial cells and cholangiocytes [3, 4]. The most common liver features in patients with COVID-19 are elevated liver aminotransferases, including aspartate aminotransferase (AST) and alanine aminotransferase (ALT), and mild elevation of alkaline gamma-glutamyl transferase (GGT). Liver biopsies of COVID-19 patients show increased mitotic cells, accompanied by eosinophilic bodies and ballooning degeneration, which are hallmarks of liver damage [5]. Notably, immune-mediated injury may be the main feature of liver injury and liver function abnormalities that occurs in patients with COVID-19. Therefore, COVID-19 can disrupt immune tolerance and lead to the occurrence of autoimmune diseases, especially autoimmune liver disease (AILD). AILDs are a group of immune disorders, including autoimmune hepatitis (AIH), primary sclerosing cholangitis (PSC), primary biliary cholangitis (PBC), and IgG4-related sclerosing cholangitis.

According to recent reports on the COVID-19 pandemic, AIH and AIH/PBC variant syndromes have emerged as a result of COVID-19 infection and vaccination [6–8]. However, more evidence is necessary to confirm this potential relationship. We collected clinical data on AILD and post-COVID-19 AILD patients to explore the relationship between COVID-19 infection and AILD, comparing the clinical and pathological characteristics to determine the differences and connections between these forms of AILD.

## 2. Materials and Methods

### 2.1. Clinical Data

The AILD group comprised 19 inpatients who were first diagnosed with AILD at Wuxi Fifth People's Hospital from January 2021 to December 2021. The post-COVID-19 AILD group comprised seven patients diagnosed with AILD after COVID-19 infection at the same hospital. These patients were recruited after December 2022, and all developed AILD within 1 month of testing positive for COVID-19.

### 2.2. Inclusion Criteria

AIH was diagnosed according to the “2019 American Association for the Study of Liver Disease (AASLD) Practice Guidelines and Guidelines: Diagnosis and Treatment of AIH in Adults and Children,” [9] and the simplified diagnostic scoring system for AIH was developed in 2008 [10]. PBC was diagnosed according to the “2018 American Association of Liver Diseases PBC Practice Guidelines” [11]. PSC was diagnosed according to the Clinical Practice Guidelines: PSC published by the American College of Gastroenterology in 2015 [12]. COVID-19 was confirmed by real-time polymerase chain reaction of throat swabs.

### 2.3. Exclusion Criteria

Exclusion criteria were as follows: (1) viral hepatitis (Hepatitis A, B, C, D, and E), drug-induced hepatitis, alcoholic liver disease, and intrahepatic bile duct stones and extrahepatic bile duct stones and other obstructive diseases; (2) severe alcoholism or intravenous drug abuse; (3) combined history of malignant tumors; (4) genetic and metabolic diseases; and (5) pregnancy.

### 2.4. Ethical Review

This study involving human participants was approved by Affiliated Wuxi Fifth Hospital of Jiangnan University, with Approval Number/ID: 2023-003-1.

### 2.5. Laboratory Examination

Routine blood tests were conducted on all patients in the two groups (including white blood cell count, red blood cell count, and hematocrit) using a fully automatic hematology analyzer (Sysmex Co., Ltd., Kobe City, Hyogo Prefecture, Japan). A fully automatic biochemical analyzer was used to assess various biochemical parameters, including alkaline phosphatase (ALP), GGT, total bilirubin (TBIL), albumin (ALB), and globin (GLB) levels. Indirect immunofluorescence was used to measure the levels of antinuclear antibodies (ANAs), anti-SM antibodies, and other serum-related antibodies. A Beckman ACL900 analyzer (Beckman Coulter, Inc., Brea, California, United States) was used to measure anticoagulation indicators, such as activated partial thromboplastin and prothrombin time. Serum immunoglobulin levels, such as immunoglobulin G (IgG), immunoglobulin M (IgM), and immunoglobulin A (IgA), were measured.

### 2.6. Pathological Examination

A total of 24 patients in the two groups underwent liver puncture biopsy. The procedures were strictly aseptic. After skin disinfection, liver puncture was performed under ultrasound guidance. Approximately 1–2 cm of liver tissue was collected from each patient. The collected liver tissue was fixed with formaldehyde (10%) and sectioned. After staining with hematoxylin–eosin (HE), at least six portal areas per specimen were assessed by microscopy, and the images were analyzed by two experienced pathologists in a double-blinded manner.

### 2.7. Immunohistochemical Staining

A ready-to-use two-step method was used to measure the expression levels of leukocyte differentiation antigen (cluster of differentiation, CD) 20, CD3, CD38, and MUM1 in the liver tissue of the two groups. Mouse anti-human CD38, mouse anti-human MUM1, mouse anti-human CD3, and mouse anti-human CD20 monoclonal antibodies were obtained from Gene Technology (Shanghai) Co., Ltd. The experiments were conducted using a Leica automatic immunohistometer (BOND III) to stain the slides. The expression levels of CD20, CD3, CD38, and MUM1 were quantified using the percentage of positive cells. For each slide, five representative high-power fields (HPFs, 400x magnification) were randomly selected. Two experienced pathologists independently counted at least 100 cells per field, recording the number of positive cells (defined by brown–yellow granular staining on the cell membrane or in the cytoplasm/nucleus). The average percentage of positive cells for each marker was calculated (number of positive cells/total cells counted × 100%). Discrepancies between the two pathologists were resolved by a third senior pathologist.

### 2.8. Statistical Analysis

Statistical analyses were conducted using SPSS 20.0 software. Normally distributed data are expressed as the mean ± standard deviation ($\overset{¯}{x}$ ± *s*), and a *t* test was used to compare two groups; nonnormally distributed data are expressed as the median (25th and 75th percentiles). The Mann–Whitney *U* test was used for comparisons between groups, and the *χ*^2^ or Fisher tests were used for comparisons between two groups. *p* < 0.05 was considered to indicate significance.

## 3. Results

### 3.1. Comparison of Clinical Characteristics Between AILD and Post-COVID-19 AILD Patients

Patients in the post-COVID-19 AILD group exhibited significantly lower age and calcium levels compared to those in the AILD group (*p* < 0.05). The IgM and ALT, and calcium levels of post-COVID-19 AILD patients were significantly greater than those of AILD patients (*p* < 0.05). No significant differences were observed between the two groups in the other routine blood and blood biochemistry indicators. In the comparison of ANA and SM antibodies, the percentage of the ANA-positive rate was significantly greater in post-COVID-19 AILD patients than in AILD patients. The difference between the two groups was significant (*p* < 0.05) (Table 1).

### 3.2. Pathological Characteristics of the Patients in the Two Groups

The liver findings were mostly normal in the early stage. Multiple acute attacks and hepatocellular necrosis result in liver shrinkage and long-term disease, leading to cirrhosis similar to that observed in chronic viral hepatitis. Liver biopsy was performed in both groups. From a macroscopic perspective, the color of liver tissue is grayish yellow and the liver tissue appears as a striated pattern, with a length ranging from 1 to 2 cm and a diameter of 0.1–1 cm. From the gross images of liver biopsies, we have found no discernible differences between the two groups.

#### 3.2.1. Microscopic Changes

AILDs, including AIH, are characterized mainly by liver cell damage. Damage to the intrahepatic bile duct system is the main pathological manifestation of PBC, PSC, and lgG4-SC. We selected 19 patients with AILD before COVID-19 infection, 11 with AIH, 5 with PBC + AIH, and 3 with AIH + PSC. Seven patients post-COVID-19 AILD were also recruited, comprising four with AIH and three with AIH + PBC.

**Figure 1:**
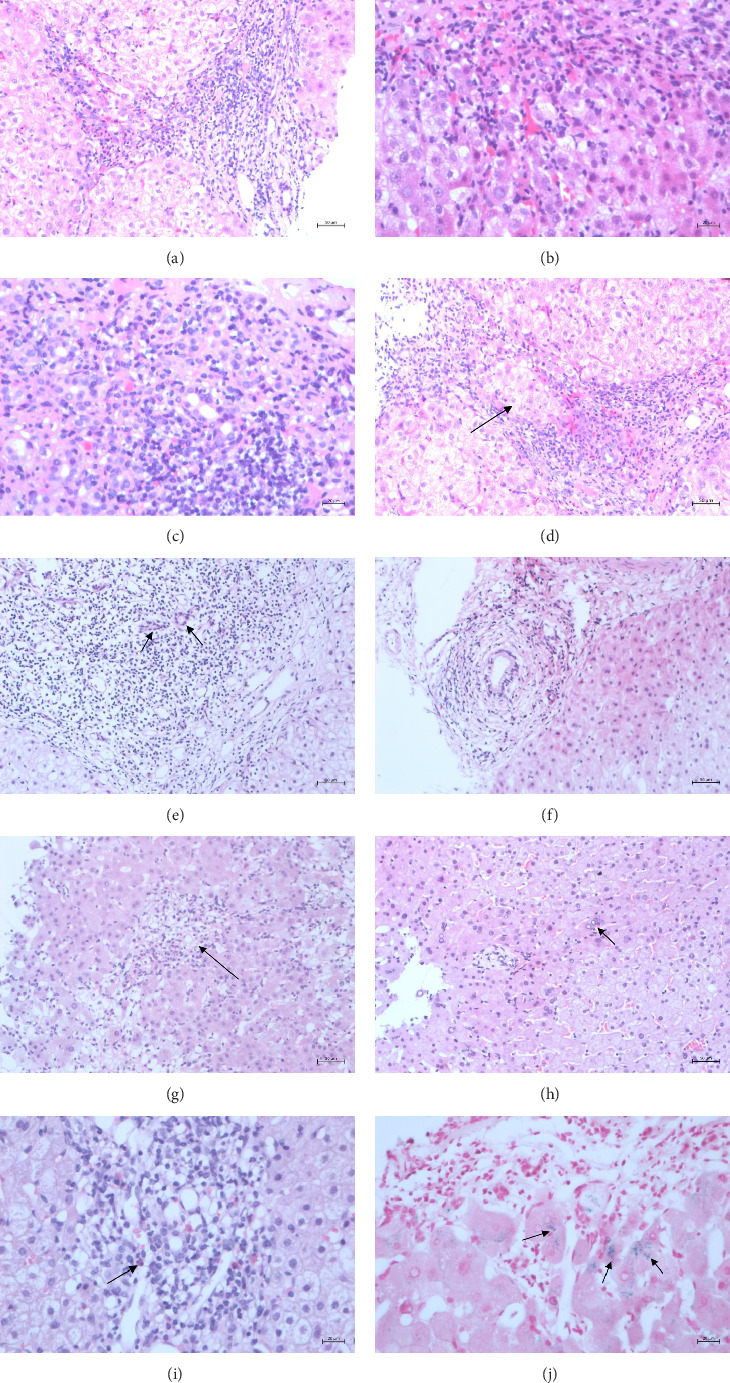
Pathological manifestations of liver tissue in patients. (a) Interface hepatitis, HE staining, post-COVID-19 AILD patients (×200); (b) interface hepatitis, HE staining, post-COVID-19 AILD patients (×400); (c) lymphocyte and plasma cell infiltration in the portal area, HE staining, post-COVID-19 AILD patients (×400); (d) rosette formation (indicated by arrows), HE staining, post-COVID-19 AILD patients (×200); (e) cholangitis (indicated by arrows), HE staining, post-COVID-19 AILD patients (×200); (f) PSC, onion-skin fibrosis of peribiliary tissue, HE staining, post-COVID-19 AILD patients (×200); (g) necrosis in the central area of hepatic lobule (zone 3), characterized by spotty necrosis of hepatocytes and infiltration of small lymphocytes and neutrophils (indicated by arrows), HE staining, post-COVID-19 AILD patients (×200); (h) multinucleated hepatocytes (indicated by arrows), HE staining, post-COVID-19 AILD patients (×200); (i) eosinophil infiltration in the portal area (indicated by arrows), HE staining, post-COVID-19 AILD patients (×400); (j) positive iron staining, special staining (indicated by arrows), post-COVID-19 AILD patients (×400).

The pathological manifestations of patients with AILD were determined. The pathological manifestations of AIH are diverse, predominantly involving liver cell damage. The main pathological characteristics of the patients were as follows:

1. Interface hepatitis (Figure 1(a)), characterized by small clusters of liver cells in the interface area, single-cell necrosis, and detachment, causing the interface of the liver lobules to demonstrate a moth-eaten shape with inflammatory cells extending from the interface into the lobules. As the disease progresses, the local hepatocyte network fibrous scaffold collapses, interstitial fibroblasts and fibroblasts proliferate, the fibrous intervals widen, and pseudolobules form, progressing to cirrhosis.
2. Lymphoplasmacytic infiltration (Figure 1(b)).
3. Liver cells in a rosette-like arrangement (Figure 1(c)). Acute and chronic inflammatory cells destroy the liver boundary plate and separate the liver cells into nest-like clusters, similar in shape to rosette nodules.
4. Penetration phenomenon (Figure 1(d)): In interface hepatitis, lymphocytes enter between liver cells. Histopathological features of PBC: Chronic nonsuppurative destructive cholangitis and loss of bile ducts can be observed (Figure 1(e)). The histopathological feature of PSC is onion-skin-like fibrosis around the intrahepatic bile ducts (Figure 1(f)).

In addition to the abovementioned pathological characteristics, the following pathological characteristics post-COVID-19 AILD were observed: necrosis in the center of the liver lobule, manifesting as liver cell punctate necrosis, small lymphocyte foci, and neutral granulocyte infiltration (Figure 1(g)); multinucleated or megakaryocytic hepatocytes (Figure 1(h)) infiltration of lymphocytes and plasma cells; increased eosinophil infiltration (Figure 1(i)); positive iron staining; and increased neutrophil infiltration at the edge of the boundary plate of the portal area (Figure 1(j)).

### 3.3. Expression of CD20, CD3, CD38, and MUM1 in the Two Groups

**Figure 2:**
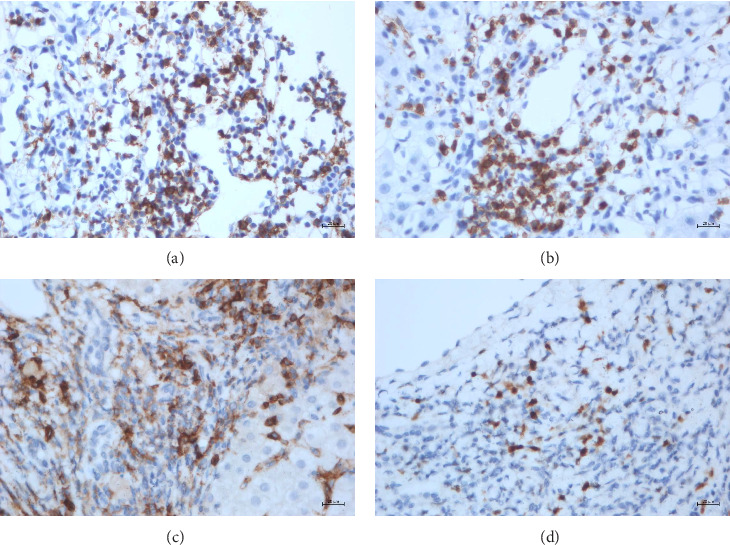
Expression of CD20, CD3, CD38, and MUM1 in liver tissues. (a) CD20 staining in patients with post-COVID-19 AILD, brown–yellow granular staining on the cell membrane (×400); (b) CD3 staining in patients with post-COVID-19 AILD, brown–yellow granular staining on the cell membrane and/or in the cytoplasm (×400); (c) CD38 staining in patients with post-COVID-19 AILD, brown–yellow granular staining on the cell membrane (×400); (d) MUM1 staining in patients with post-COVID-19 AILD, brown–yellow granular staining in the cell nucleus (×400).

CD20 expression was considered positive when brown–yellow particles were observed on the cell membrane of the lymphocytes (Figure 2(a)). CD3 expression was considered positive when brown–yellow particles were observed on the lymphocyte cell membrane or in the cytoplasm (Figure 2(b)). Brown–yellow particles on the lymphocyte cell membrane indicated positive expression of CD38 (Figure 2(c)). Brown–yellow particles in the cell nucleus or lymphocyte cytoplasm indicated positive expression of MUM1 (Figure 2(d)).

The immunohistochemistry results showed that the CD20 level in post-COVID-19 AILD patients was greater than that in those with AILD, while the MUM1 level was lower in the AILD group (*p* < 0.05) (Table 2).

## 4. Discussion

AILD is the second most common cause of chronic liver disease and has gradually become a prominent concern in the liver disease field. The incidence of AILD is increasing annually in China [13]. AILD is characterized by increased inflammation and progressive liver fibrosis. Untreated AILD can be life-threatening, and patients may ultimately require a liver transplant. Therefore, the early diagnosis and treatment of AILD are crucial. The three major categories of AILD are AIH, PBC, and PSC. PBC is the most common form. In recent years, PBC has ranked second in indications for adult liver transplantation, and the associated mortality rate is 1.6%–2% among patients with cirrhosis. PBC is more common in Eastern than Western countries, with annual incidence rates of 4.3/100,000 and 0.86/100,000 people in the United States and South Korea, respectively. The prevalence of PBC in the United States and South Korea is 39.2 and 47.5 per 100,000 people, respectively, and it usually occurs in women in their 50s and 60s. PBC is diagnosed according to elevated serum ALP, positive anti-mitochondrial antibodies, and nonsuppurative destructive cholangitis [14]. The global annual incidence rate of AIH is 1.37/100,000 people, which is similar to those in Asia, Europe, and the United States. The male-to-female ratio is 1:5. The global prevalence of AIH is 17.44/100,000 people, with large regional differences [15]. The average age at onset of AIH is > 50 years [16]. PSC is a chronic cholestatic disease of unknown etiology characterized by the destruction of bile ducts due to inflammation and fibrosis. The prevalence of PSC in Northern Europe and North America is 4.15/100,000 and 16.2/100,000, respectively, and the prevalence in Japan is 0.95/100,000. PSC mainly occurs in men, and 50%–80% of PSC patients have ulcerative colitis.

The mechanism by which viral infections disrupt immune tolerance and trigger autoimmunity, often referred to as “viral mimicry,” is not unique to SARS-CoV-2. Other viruses, such as Epstein–Barr virus (EBV), hepatitis C virus (HCV), and cytomegalovirus (CMV), have also been implicated in the pathogenesis of various autoimmune disorders. EBV infection is associated with systemic lupus erythematosus, multiple sclerosis, and rheumatoid arthritis [17]. Post-COVID-19 AILD shares similarities with AILD triggered by other viruses in multiple aspects, including clinical manifestations and serological characteristics, such as a high positivity rate of ANAs and elevated IgM levels. This suggests the existence of a common viral-induced pathway for autoimmunity.

AILD is an autoaggressive immune response involving interactions between T-regulated lymphocytes or plasma cells and specific or nonspecific antigen targets. During the liver autoimmune reaction, “self”-antigens directly or indirectly activate NKT cells and other innate immune cells via antigen-presenting cells. Viral infections are considered potential environmental causes of AILD because the immune system can recognize viral peptides that imitate self-antigens through a mechanism known as “molecular mimicry,” [18] resulting in autoimmune tolerance [19]. COVID-19 infection can cause immune tolerance, leading to a new onset or outbreak of AILD. Exploring the underlying mechanism of AILD in patients with COVID-19–associated pneumonia has certain significance. We found that many cases of new AILD occurred within 1 month after COVID-19 diagnosis, especially within 2–4 weeks. These patients demonstrated typical clinical manifestations of AILD, such as fatigue, jaundice, and skin itching, which attracted the attention of physicians and led to the diagnosis of AILD through follow-up examinations. We compared the clinical and pathological characteristics of patients with AILD and with post-COVID-19 AILD to compare these two presentations. The characteristics of post-COVID-19 AILD were identified, providing a basis for the early detection of this disease.

Several studies have shown that serological indicators and liver function in patients with COVID-19 are increased to varying degrees, mainly manifesting as increased levels of ALT and AST, accompanied by a slight increase in TB [20–23]. The incidences of elevated ALT and AST range from 2.5% to 50.0% and 2.5%–61.1%, respectively. Sun et al. showed that the elevated transaminase rate among young people with COVID-19 pneumonia ranges from 14% to 53% [24]. COVID-19 can directly infect liver cells that express the receptor for the virus. In a meta-analysis examining 47 studies, including 10,890 patients, the rates of ALT and AST elevation were 15.0% and 20%, respectively [25]. In a case series including 1100 people infected with COVID-19, AST and ALT levels were greater in severe cases (56% and 28%, respectively) than in mild-moderate cases (18% and 29%, respectively), and AST levels were associated with mortality. AST is present in the cytoplasm and mitochondria, and AST release is increased due to viral damage to mitochondria. In a meta-analysis of liver injury among patients with COVID-19, it was found that the overall global prevalence of elevated AST, ALT, TBIL, GGT, and ALP was 23.2%, 21.2%, 9.7%, 15.0%, and 4.0%, respectively. Approximately 25% of patients with COVID-19 had elevated liver enzymes [26], and almost all patients had normal ALP levels. There was no significant increase in bilirubin or ALP levels in patients with COVID-19. Comparing the liver functions of the two groups revealed that the AST level of those with post-COVID-19 AILD was significantly greater than that of the AILD group, suggesting that the AILD-related liver function damage following COVID-19 is more serious, potentially related to the severe infection of liver cells with COVID-19. Thus, COVID-19 increases the effect of liver injury.

The COVID-19 capsid spike (S) glycoprotein enters the surface of Type 2 pneumocytes through ACE2. A transmembrane serine protease (2TMPRSS2) helps prime the spike protein and facilitates its entry into host cells. Once inside the host cell, the virus replicates and releases virions to infect other ACE2-expressing cells [27]. Two independent cohort studies revealed that ACE2 is expressed in 2.6% of hepatocytes and 59.7% of cholangiocytes [28], suggesting that COVID-19 may directly bind to ACE2-positive cholangiocytes, causing liver dysfunction. Considering the expression of ACE2 receptors in liver cells, COVID-19 infection might cause more severe liver function damage, explaining why AILD-related liver function damage of COVID-19 infection is more serious.

The different types of AILDs have different sex-based tendencies. AIH is characterized by female dominance (female:male = 7:1). PSC is considered an autoimmune disease with atypical characteristics, with male dominance (female:male = 1:2). The most common AILD, PBC, mainly affects women. It is possible that because the liver tissue of PBC patients highly expresses estrogen receptors, estrogen in female patients binds to highly expressed estrogen receptors in liver tissue, activating these receptors; estrogen can promote the Th1 cell response and increase susceptibility to autoimmune diseases induced by T lymphocytes [29]. This mechanism might explain the high proportion of women among AILD patients. In this study, there was no significant difference in sex between the AILD group and the post-COVID-19 AILD group. However, women were dominant in both groups. The incidence rate in women was significantly greater than that in men, and the affected women were mainly middle-aged. This result is similar to those presented in previous reports related to AILD [30].

An important feature of AILD is the presence of specific and nonspecific ANAs in the serum. The components of the nucleus and cytoplasm are the target antigens of ANAs, first discovered in systemic lupus erythematosus patients [31]. Given that ANAs can also be detected in healthy people or patients with other liver diseases, such as fatty liver disease, drug-induced liver injury, viral hepatitis, or other autoimmune diseases, they are not specific to AIH. ANAs can bind with cell membrane proteins and initiate cytotoxicity, destroying liver cells and leading to inflammatory liver necrosis, the destruction of the normal structure of the liver lobules, the reduction of liver metabolic function, and the promotion of fibroblast proliferation and collagen synthesis. These processes can lead to liver fibrosis, thereby inducing AILD. In this study, 10.52% of AILD patients and 57.14% of post-COVID-19 AILD patients were positive for ANAs, consistent with the characteristics of AILD. ANAs are of substantial significance in AILD diagnosis. The positivity rate in post-COVID-19 AILD patients is relatively high, indicating that the COVID-19 virus has a significant cytotoxic effect on the body, causing obvious autoantibody production. It is important to note that detecting these antibodies is insufficient for diagnosis because they are nonspecific and occasionally demonstrate low sensitivity. Additionally, antibody titers vary over the disease course. Since diagnosing AILD requires considering multiple parameters, pathologists and clinicians should be aware of the importance of ANAs. The presence of antibodies does not necessarily imply the presence of disease, but physicians should always interpret AILD according to clinical, biochemical, and histological parameters.

AILD is a primary, endogenous pathological change in the liver. The onset is difficult to detect and cannot be diagnosed through viral testing. Liver biopsy is the gold standard for diagnosing various liver diseases and has important clinical value in the diagnosis, differential diagnosis, and treatment of AILD. Liver biopsy and corresponding histological examination are important methods for diagnosing AILD. Characteristic cytological changes in the immune response can be observed in liver tissue. Patients with AIH mainly exhibit inflammatory necrosis of hepatocytes and PBC. The typical histological feature is interface hepatitis, characterized by inflammatory cells that extend into the lobules, causing the necrosis or loss of adjacent hepatocytes. Other nonspecific features suggestive of AILD include lymphocyte infiltration and hepatocellular rosettes [32]. If only one of the above manifestations is present, a specific diagnosis of AIH cannot be made; however, the simultaneous presence of the three manifestations is considered a characteristic manifestation of AIH. PBC mainly manifests as granulomatous and lymphocytic cholangitis, with bile duct opening and nonsuppurative destruction of the small bile ducts, leading to biliary liver fibrosis and cirrhosis. PSC exhibits portal area expansion, diffuse lymphocyte infiltration in the portal area, bile duct epithelial degeneration, peribiliary inflammation with onion skin-like changes, and bile stasis in surrounding hepatocytes, gradually developing into liver fibrosis. In this study, moderate to severe interface hepatitis was detected in the liver tissue of AIH patients, but no bile duct lesions were detected. Bile duct lesions were observed in PBC patients and were accompanied by mild interface inflammation, piecemeal necrosis, or both. The pathology observed in this group is a typical manifestation of AILD. In summary, immunological indicators such as ANAs may be used to diagnose AILD initially, and a combination of liver histological and pathological examination can help confirm the diagnosis.

AILD demonstrates different features depending on its classification. The onset of AILD is difficult to detect, and the main manifestations of the disease are similar to those of acute viral hepatitis. The most common symptom is fatigue. Common clinical manifestations include itching, jaundice, anorexia, nausea, and an aversion to greasy foods. Fatigue, anorexia, and liver pain are also primary symptoms [33]. The main clinical symptoms of the patients in our study were jaundice, itching, occasional fatigue, and anorexia, all of which arose within 1 month after testing positive for COVID-19. Most patients were admitted to the hospital as a result of the typical clinical manifestations. The most common sign of AILD is hepatomegaly. Splenomegaly can occur in patients with and without cirrhosis. Patients may have symptoms similar to cirrhosis, such as spider nevi, often found on the face, neck, and arms. Since COVID-19 has an acute onset and the duration of onset is relatively short, no special signs have been found to date.

Moreover, AILD is closely related to immune dysfunction, and IgG and IgM are important molecules that mediate humoral immunity. Immunoglobulin has received increasing attention in the study of AILD patients. Immunoglobulin is of great value in diagnosing AILD, and relevant evidence can be found through serology and histology. In this study, the IgM concentration was found to be increased and was significantly greater in the post-COVID-19 AILD group than in the AILD group. The difference was significant (*p* < 0.05), suggesting that COVID-19 may worsen or provoke the humoral immune dysfunction in underlying AILD. This study confirmed that immunoglobulins play an irreplaceable role in diagnosing AILD. Hypocalcemia is relatively common in autoimmune diseases, which may be related to mechanisms such as immune cell activation and cytokine release. Its level changes may reflect the activity of the disease, indicating that after COVID-19 infection, the patient's disease status, activity status, and immune status all undergo corresponding changes.

CD20 can act on B cells by regulating transmembrane calcium ions and plays a role in B-cell proliferation and differentiation. MUM1 is a lymphoid-specific transcription factor encoded by the MUM1 gene and a member of the interferon regulatory factor family that can regulate the maturation and differentiation of B cells into plasma cells. MUM1 immunostaining has been used to assess plasma cell precursors in AIH [34]. Among those with AILD, the number of MUM1-positive cells is highest in PBC patients [35]. Plasma cells produce antibodies, exert humoral immune effects, and stimulate the body to produce cellular immunity, causing autoimmune diseases. Although CD20 and MUM1 are both B-cell markers, CD20 is not expressed in plasma cells, and MUM1 is expressed in the terminal stage of B cells. In this study, CD20 levels were higher in the post-COVID-19 AILD group than in the AILD group, suggesting that CD20 is involved in post-COVID-19 AILD. Other studies have shown that CD38 and MUM1 are significantly related to liver cirrhosis [36]. The MUM1 level was lower in the post-COVID-19 AILD group than in the AILD group, suggesting that patients with post-COVID-19 AILD have not yet developed cirrhosis, a finding related to the acute onset and rapid development of the disease.

However, this study has certain limitations, which is the relatively small sample size; particularly in the post-COVID-19 AILD group, it has only seven cases. This limitation is primarily due to the fact that post-COVID-19 AILD is a newly recognized and relatively rare condition. It has the challenge of recruiting a large number of such cases within a specific time. The limited number of cases may affect the statistical power and the generalizability of the results. Larger, multicenter prospective studies are needed to confirm these observations and to further elucidate the distinct clinical and pathological features of AILD following COVID-19.

In summary, after comparing the clinical characteristics of the AILD and post-COVID-19 AILD groups, we found that liver function damage of post-COVID-19 AILD is more serious, with obvious characteristics of an acute attack. Physicians should pay attention to fatigue and anorexia in those diagnosed with COVID-19 and consider AILD, using ANA examination and pathological features for diagnosis.

## Figures and Tables

**Table 1:** Comparison of clinical characteristics between AILD and post-COVID-19 AILD patients.

Item	Reference value	AILD (*n* = 19) mean ± SD or median (IQR)	Post-COVID-19 AILD (*n* = 7) mean ± SD or median (IQR)	*t*/*Z*/*χ*^2^ value	*p* value
Age (years, mean ± SD)	—	57.26 ± 8.97	46.57 ± 14.11	2.304	0.030

Sex/*n* (%)	Male	3 (15.8)	2 (28.6)	0.538	0.463
	Female	16 (84.2)	5 (71.4)		

Hemoglobin (g/L, mean ± SD)	130–175	116.63 ± 15.08	123.57 ± 16.98	−1.008	0.324

Hematocrit (mean ± SD)	0.40–0.50	0.36 ± 0.04	0.37 ± 0.05	−0.956	0.349

Red blood cell (×10^12^/L, median (IQR))	4.3–5.8	3.92 (3.50–4.16)	4.07 (3.69–4.27)	−1.208	0.259

Leucocyte (×10^9^/L)	3.5–9.5	4.22 ± 1.86	4.59 ± 0.42	−0.802	0.431

Thrombocyte (×10^9^/L)	125–350	134.11 ± 72.44	158.14 ± 40.32	−1.066	0.299

Mean corpuscular volume (fL)	82–100	96.07 ± 5.54	93.44 ± 1.59	1.222	0.234

Red blood cell distribution width (%)	12.2–17.8	13.99 ± 1.78	14.17 ± 2.00	−0.217	0.830

Sodium (mmol/L)	137–147	140.36 ± 3.37	139.16 ± 3.84	0.777	0.445

Potassium (mmol/L)	3.5–5.3	3.82 ± 0.33	3.59 ± 0.51	1.319	0.200

Chloride (mmol/L)	96–108	106.53 ± 3.24	106.40 ± 2.50	0.097	0.924

Blood sugar (mmol/L)	4.1–5.9	4.91 (4.60–6.50)	4.47 (3.99–4.90)	−1.909	0.056

Creatinine (μmol/L)	57–97	52.00 (43.00–69.20)	64.00 (40.00–67.00)	−0.260	0.795

Calcium (mmol/L)	2.18–2.60	2.30 (2.24–2.52)	2.20 (2.07–2.22)	−2.779	0.005

Total protein (g/L)	65–85	67.29 ± 7.31	65.200 ± 8.648	0.618	0.542

Albumin (g/L)	40–55	36.91 ± 4.79	33.33 ± 4.61	1.705	0.101

Alb/glb ratio	1.5–2.5	1.20 (1.12–1.46)	1.12 (0.62–1.24)	−1.158	0.247

Total bilirubin (μmol/L)	3.42–20.52	21.00 (12.80–71.00)	38.00 (23.20–43.90)	−0.954	0.340

Direct bilirubin (μmol/L)	0–5.1	9.40 (5.60–51.40)	21.60 (11.30–27.70)	−0.838	0.402

Alanine aminotransferase (ALT, U/L)	10–49	81.00 (43–230)	109.00 (66.50–314.00)	−0.781	0.435

Aspartic acid transferase (AST, U/L)	0–34	83.00 (34.00–240.00)	126.00 (81.50–329.40)	−0.781	0.019

Glutamyltransferase (GGT, U/L)	0–73	154.00 (100–231.8)	101.00 (92.00–189.10)	−1.070	0.285

Prothrombin time (s)	11.5–15.5	13.40 (12.8–14.3)	13.60 (12.30–14.50)	−0.174	0.862

Partial thromboplastin time (s)	26–40	40.26 ± 6.38	38.93 ± 3.53	0.521	0.607

HA-Ab-IgM/*n* (%)	Negative	19 (100.0)	7 (100.0)	≤ 0.001	0.083
	Positive	0 (0.0)	0 (0.0)		

HBsAg/*n* (%)	Negative	19 (100.0)	7 (100.0)	≤ 0.001	0.083
	Positive	0 (0.0)	0 (0.0)		

HBcAb-IgM/*n* (%)	Negative	19 (100.0)	7 (100.0)	≤ 0.001	0.083
	Positive	0 (0.0)	0 (0.0)		

HC-Ab-IgG/*n* (%)	Negative	19 (100.0)	7 (100.0)	≤ 0.001	0.083
	Positive	0 (0.0)	0 (0.0)		

HE-Ab-IgM/*n* (%)	Negative	19 (100.0)	7 (100.0)	≤ 0.001	0.083
	Positive	0 (0.0)	0 (0.0)		

HD-Ab-IgM/*n* (%)	Negative	19 (100.0)	7 (100.0)	≤ 0.001	0.083
	Positive	0 (0.0)	0 (0.0)		

IgG (g/L)	7.51–15.60	13.80 (13.2–19.1)	24.20 (12.70–38.60)	−1.562	0.118

IgM (g/L)	0.46–3.04	1.442 ± 0.877	2.349 ± 0.768	−2.410	0.024

IgA (g/L)	0.82–4.53	3.00 (2.22–3.65)	2.77 (1.55–3.38)	−0.780	0.435

Anti-SM antibodies/*n* (%)	Negative	7 (36.8)	4 (57.1)	0.864	0.312
	Positive	12 (63.2)	3 (42.9)		

Antinuclear antibodies (ANAs)/*n* (%)	Negative	17 (89.5)	3 (43.9)	12.831	≤ 0.001
	Positive	2 (10.5)	4 (57.1)		

*Note:* Continuous variables with normal distribution are presented as mean ± standard deviation and compared using the independent samples *t* test; those with non-normal distribution are presented as median (interquartile range) and compared using the Mann–Whitney *U* test. Categorical variables are presented as number (*n*) and compared using the chi-square test or Fisher' exact test, as appropriate. A *p* value < 0.05 was considered statistically significant.

**Table 2:** Expression of CD20, CD3, CD38, and MUM1 in the two groups of patients (%).

	CD20	CD3	CD38	MUM1
AILD group (*n* = 19)	20.000 ± 12.748	68.235 ± 14.678	15.588 ± 8.993	20.588 ± 8.269
Post-COVID-19 AILD (*n* = 7)	37.143 ± 28.115	54.286 ± 24.398	20.000 ± 10.000	12.143 ± 7.559
*t* value	−2.089	1.739	−1.059	2.327
*p* value	0.048	0.096	0.301	0.030

## Data Availability

The data that support the findings of this study are available on request from the corresponding author, Juanjuan Fu, upon reasonable request.
